# Fourth-Generation Progestins Inhibit 3β-Hydroxysteroid Dehydrogenase Type 2 and Modulate the Biosynthesis of Endogenous Steroids

**DOI:** 10.1371/journal.pone.0164170

**Published:** 2016-10-05

**Authors:** Renate Louw-du Toit, Meghan S. Perkins, Jacky L. Snoep, Karl-Heinz Storbeck, Donita Africander

**Affiliations:** Department of Biochemistry, University of Stellenbosch, Private Bag X1, Matieland, 7602, South Africa; State University of New York Upstate Medical University, UNITED STATES

## Abstract

Progestins used in contraception and hormone replacement therapy are synthetic compounds designed to mimic the actions of the natural hormone progesterone and are classed into four consecutive generations. The biological actions of progestins are primarily determined by their interactions with steroid receptors, and factors such as metabolism, pharmacokinetics, bioavailability and the regulation of endogenous steroid hormone biosynthesis are often overlooked. Although some studies have investigated the effects of select progestins on a few steroidogenic enzymes, studies comparing the effects of progestins from different generations are lacking. This study therefore explored the putative modulatory effects of progestins on *de novo* steroid synthesis in the adrenal by comparing the effects of select progestins from the respective generations, on endogenous steroid hormone production by the H295R human adrenocortical carcinoma cell line. Ultra-performance liquid chromatography/tandem mass spectrometry analysis showed that the fourth-generation progestins, nestorone (NES), nomegestrol acetate (NoMAC) and drospirenone (DRSP), unlike the progestins selected from the first three generations, modulate the biosynthesis of several endogenous steroids. Subsequent assays performed in COS-1 cells expressing human 3βHSD2, suggest that these progestins modulate the biosynthesis of steroid hormones by inhibiting the activity of 3βHSD2. The K_i_ values determined for the inhibition of human 3βHSD2 by NES (9.5 ± 0.96 nM), NoMAC (29 ± 7.1 nM) and DRSP (232 ± 38 nM) were within the reported concentration ranges for the contraceptive use of these progestins *in vivo*. Taken together, our results suggest that newer, fourth-generation progestins may exert both positive and negative physiological effects via the modulation of endogenous steroid hormone biosynthesis.

## Introduction

Synthetic progestogens (progestins), were developed to have similar progestogenic properties, but greater bio-availabilities, half-lives and potencies than the rapidly metabolized natural progestogen, progesterone (Prog) [1, 2]. Progestins are mostly derived from parent compounds such as Prog and testosterone (reviewed in [3]), with those structurally related to Prog referred to as 17α-hydroxyprogesterone (17OH-Prog) and 19-norprogesterone derivatives, and those related to testosterone known as 19-nortesterone derivatives. A variety of these structurally diverse compounds are available, and are classified into four consecutive generations. Like Prog, these progestins mediate their biological effects by binding to the progesterone receptor (PR), and are used in many applications in female reproductive medicine including contraception and hormone replacement therapy (HRT) [4, 5] (reviewed in [3, 6]). A number of side-effects have however been reported with their clinical use and include weight gain, acne, increased risk of invasive breast cancer, cardiovascular disease (CVD) and modulation of immunity in the female genital tract (reviewed in [6]).

To date it has been suggested that the mechanism underlying most of these adverse effects are most likely due to some progestins interacting with steroid receptors other than the PR [7–13]. Thus, the newer, fourth-generation progestins were developed to be “purer” progestogens by having stronger affinities for the PR. Although these progestins may also bind to other steroid receptors, their activities are similar to the natural PR ligand, Prog, in that they are devoid of estrogenic, androgenic, glucocorticoid and mineralocorticoid activity, with some, like Prog, eliciting anti-androgenic and/or anti-mineralocorticoid effects [5, 14] (reviewed in [3, 6]). However, some recent studies indicate that these newer generation progestins also display adverse effects. For example, the risk of developing venous thromboembolism (VTE) has been shown to increase with the use of combined oral contraceptives (COC) containing the fourth-generation progestin drospirenone (DRSP) [15–17].

**Fig 1:**
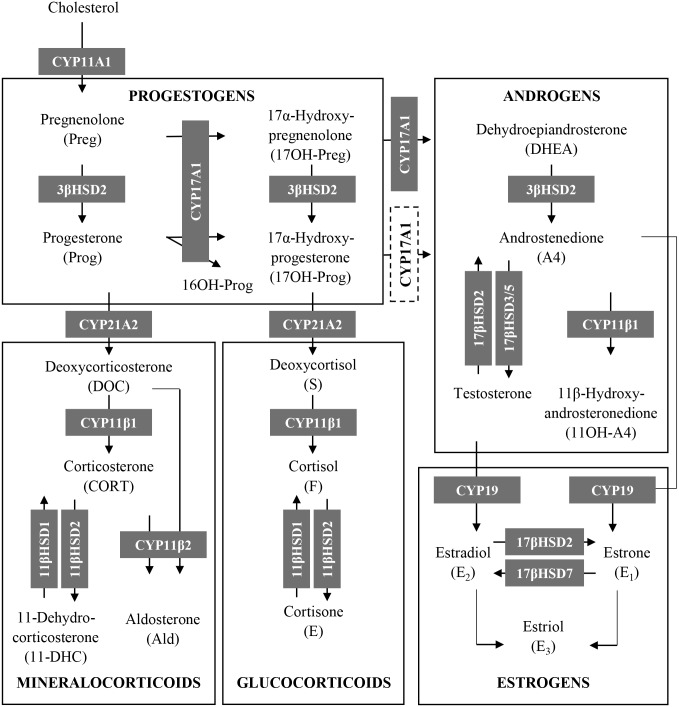
The biosynthesis of human steroid hormones consists of multiple reactions which are catalysed by specific steroidogenic enzymes (grey boxes). The conversion of 17α-hydroxyprogesterone (17OH-Prog) to androstenedione (A4) by CYP17A1 is shown as a dashed box as 17OH-Prog is a poor substrate for the 17,20-lyase activity of human CYP17A1 [22, 31].

This raises the possibility that a mechanism other than off-target steroid receptor-mediated effects may be involved. One possibility, and an area of research that has received little attention, is the influence of progestins on adrenal steroid biosynthesis. It is well documented that abnormal hormone levels due to the modulation of adrenal steroidogenesis are associated with numerous undesirable conditions [18–21] (reviewed in [22]). The limited number of studies that have in fact investigated the effects of progestins on adrenal steroid biosynthesis in humans have primarily focussed on the first-generation progestin, medroxyprogesterone acetate (MPA), and showed a reduction in the serum levels of the endogenous glucocorticoid cortisol [23–26], the endogenous androgen precursors androstenedione (A4) and dehydroepiandrosterone sulphate (DHEA-S) [25], and the endogenous androgen testosterone [27]. Recent studies examining the effects of progestins developed after the first generation, such as levonorgestrel (LNG), nomegestrol acetate (NoMAC) and DRSP, also showed decreased concentrations of androgens and their precursors [28–30]. The biosynthesis of steroid hormones are dependent on the function of steroidogenic enzymes, which consists of substrate-selective cytochrome P450 enzymes (CYP’s) and hydroxysteroid dehydrogenases (HSD’s) (Fig 1) (reviewed in [22, 31–33]). Interestingly, only a few studies have examined the influence of progestins on the activity of these enzymes, and most of the studies focus on the effects of MPA. For example, MPA has been shown to inhibit the activity of both human [34] and rat [35, 36] 3β-hydroxysteroid dehydrogenase (3βHSD), while suppressing the activity of rat, but not human, cytochrome P450 17α-hydroxylase/17,20 lyase (CYP17A1) [34, 36, 37]. Although some studies have investigated the effects of other progestins, such as norethisterone (NET) and LNG, on the activity and/or mRNA expression of steroidogenic enzymes, these studies are limited to rat [38] and fish [39] models. Considering that different species express different enzyme isoforms, which have different functions and substrate specificities [40–42], it is probable that the effects of progestins in animal models will not reflect their actions on human enzymes. It is thus clear that studies investigating the effects of progestins on human steroidogenic enzymes are needed, and more so, a direct comparative study of the influence of progestins from the different generations.

**Fig 2:**
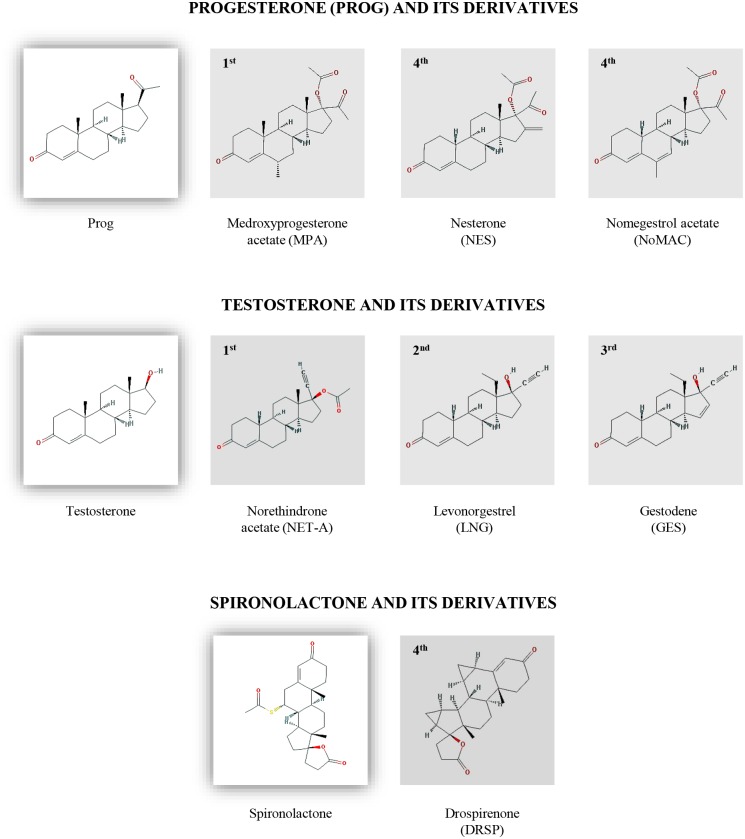
Chemical structures of the endogenous steroids progesterone (Prog) and testosterone, the synthetic MR antagonist spironolactone, and the progestins used in this study : Medroxyprogesterone acetate (MPA), nestorone (NES), nomegestrol acetate (NoMAC), norethisterone/norethindrone acetate (NET-A), levonorgestrel (LNG), gestodene (GES) and drospirenone (DRSP). The inserts (1^st^, 2^nd^, 3^rd^ and 4^th^) denote the four consecutive generations of progestins.

The present study thus directly compared the effects of select progestins from different generations on the biosynthesis of steroids by the H295R human adrenocortical carcinoma cell line, which expresses all the steroidogenic enzymes required for the biosynthesis of progestogens, mineralocorticoids, glucocorticoids and adrenal androgen [43–46]. The comparison included the first-generation progestins MPA and NET acetate (NET-A), the second-generation progestin LNG, the third-generation progestin gestodene (GES) and the fourth-generation progestins nestorone (NES), NoMAC and DRSP (Fig 2). Specifically, we used ultra-performance liquid chromatography/tandem mass spectrometry (UPLC–MS/MS) to measure not only the end products of the progestogenic, mineralocorticoid, glucocorticoid and androgenic pathways, but also to identify the steroid intermediates which are affected by the progestins. Furthermore, we also determined whether the progestins themselves are metabolized in the H295R cell line. Our results indicate that fourth-generation progestins modulate endogenous steroid biosynthesis due to the inhibition of human 3βHSD2 and/or CYP17A1 activity. Moreover, we determined inhibition constant (K_i_) values for 3βHSD2 in the nanomolar range for NES, NoMAC and DRSP, with the mechanisms of inhibition best fitted to the experimental data indicating that NES and DRSP are non-competitive inhibitors of 3βHSD2, while NoMAC is a competitive inhibitor of this enzyme.

## Materials and Methods

### Test compounds and standards

MPA, NET-A, LNG, GES, NES, NoMAC, DRSP, pregnenolone (Preg), Prog, 17OH-Preg, 17OH-Prog, 16OH-Prog, deoxycorticosterone (DOC), corticosterone (CORT), 11-dehydrocorticosterone (11-DHC), aldosterone (Ald), deoxycortisol, cortisol, cortisone, dehydroepiandrosterone (DHEA), A4, testosterone, forskolin (FSK) and trilostane, were obtained from Sigma–Aldrich, South Africa, while 11β-hydroxyandrostenedione (11OH-A4) was purchased from Steraloids, USA. All test compounds, as well as FSK, were prepared in dimethylsulfoxide (DMSO), and added to the culturing medium at a final concentration of 0.2% DMSO. The deuterated internal standards, d2-testosterone, d9-Prog, d9-17OH-Prog and d4-cortisol were purchased from Cambridge Isotope Laboratories (Maryland, USA).

### Plasmids

The plasmids expressing human 3βHSD2 (pCDNA6-hHSD3β2-V5), CYP17A1 (pIRES-hCYP17A1-V5-X-hCYPB5-6HIS), and CYP21A2 (pCDNA6-hCYP21A2-V5) were generous gifts from Prof Wiebke Arlt (Institute of Metabolism and Systems Research, University of Birmingham, UK). Plasmid DNA was purified using the NucleoBond^®^ Xtra Maxi kit (Machery-Nagel GmbH, Germany) according to the manufacturer’s instructions.

### Cell culture

The human H295R adrenocortical carcinoma cell line was a generous gift from Prof William E. Rainey (University of Michigan, Medical School, Molecular and Integrative Physiology, USA), and was cultured as previously described [47]. The COS-1 monkey kidney cell line was purchased from the American Type Culture Collection (ATCC) and cultured as previously described [12]. To ensure that only mycoplasma-negative cells were used in experiments, cell cultures were regularly tested for mycoplasma infection using Hoechst staining [48].

### Steroid biosynthesis and progestin metabolism in the H295R cell line

H295R cells were seeded into 12-well plates at 4 x 10^5^ cells per well, and two days later treated with DMSO (vehicle control) or 1 μM MPA, NET-A, LNG, GES, NES, NoMAC or DRSP, in the absence and presence of 10 μM FSK. As a negative control, medium containing the test compounds were added to 12-well plates (no cells) and incubated at 37°C in an atmosphere of 90% humidity and 5% CO_2_. After 48 hours, the medium (500 μl) was removed and steroids extracted using a 10:1 volume of dichloromethane to culture medium as described previously [47]. Briefly, 15 ng of the internal standards, d2-testosterone, d4-cortisol, d9-Prog and d9-17OH-Prog, were added to the samples, vortexed for 10 minutes and centrifuged at 3 000 rpm for 5 minutes. The dichloromethane phase containing the steroids were transferred to clean test tubes and dried at 50°C under nitrogen. The dried steroid residue was resuspended in 200 μl 50% methanol, vortexed for 2 minutes and stored at -20°C prior to analysis by UPLC–MS/MS. The cells were washed with 1x PBS, lysed with passive lysis buffer (0.2% (v/v) Triton, 10% (v/v) glycerol, 2.8% (v/v) TRIS-phosphate-EDTA and 1.44 mM EDTA) and the total protein concentration determined using the Bradford protein assay method [49]. All experiments were performed in parallel under the same experimental conditions.

### H295R cell viability

The colorimetric MTT (3-(4,5-dimethylthiazolyl-2)-2,5-diphenyltetrazolium bromide) assay was used, and performed essentially as previously described in [50] with the following modifications. Briefly, H295R cells were plated into 96-well plates at a cell density of 1 × 10^4^ cells per well, and treated for 48 hours with DMSO (vehicle control) or 1 μM test compound in the absence or presence of 10 μM FSK. Four hours prior to the end of the incubation period, the medium was aspirated and replaced with 150 μl DMEM/F12 supplemented with 0.1% cosmic calf serum (HyClone^®^ Thermo Scientific Inc., USA), 100 IU/ml penicillin and 100 μg/ml streptomycin (Sigma-Aldrich, South Africa) and 0.01% gentamycin (Gibco, Paisley, UK) and 5 mg/ml of the MTT solution (Sigma-Aldrich, South Africa). At the end of the incubation period, the medium was aspirated and the crystals resuspended in 200 μl solubilisation solution (DMSO). The plates were covered with foil and incubated at room temperature for 5 minutes with agitation, followed by the absorbance measurement at 550 nm using a BioTek^®^ PowerWave 340 spectophotometer.

### Steroid conversion assays in transiently transfected COS-1 cells

COS-1 cells were seeded into 10 cm dishes at 2 x 10^6^ cells per dish. On day 2, the cells were transiently transfected with 7.5 μg of the appropriate expression vector for human 3βHSD2 (pCDNA6-hHSD3β2-V5), CYP17A1 (pIRES-hCYP17A1-V5-X-hCYPB5-6HIS) or CYP21A2 (pCDNA6-hCYP21A2-V5), using the X-tremeGENE 9 DNA transfection reagent (Roche Molecular Biochemicals, South Africa) in accordance with the manufacturer’s instructions. After 24 hours, the cells were replated into 24-well plates at a density of 1 × 10^5^ cells per well, and incubated for 72 hours. To assay for the inhibition of substrate conversion by the progestins, the cells were treated with the appropriate steroid substrate, 1 μM Preg (for 3βHSD2) or Prog (for CYP17A1 and CYP21A2) or 17OH-Prog (CYP21A2), in the absence or presence of 1 μM MPA, LNG, GES, NES, NoMAC or DRSP. The duration of hormone treatment was based on optimal substrate conversion assays in COS-1 cells. Following the optimal treatment time, 500 μl of the medium was removed, the steroids/progestins extracted and the samples prepared for UPLC-MS/MS analysis as described in above. The cells were washed with 1x PBS, lysed with passive lysis buffer (0.2% (v/v) Triton, 10% (v/v) glycerol, 2.8% (v/v) TRIS-phosphate-EDTA and 1.44 mM EDTA) and the total protein concentration determined using the Bradford protein assay method [49].

### Kinetic analysis in transiently transfected COS-1 cells

COS-1 cells were seeded into 10 cm dishes at 2 x 10^6^ cells per dish. On day 2, the cells were transiently transfected with 7.5 μg of the expression vector for human 3βHSD2 (pCDNA6-hHSD3β2-V5) using the X-tremeGENE 9 DNA transfection reagent (Roche Molecular Biochemicals, South Africa) in accordance with the manufacturer’s instructions. After 24 hours, the cells were replated into 24-well plates at a density of 5 × 10^4^ cells per well, and incubated for 48 hours. The cells were subsequently treated with Preg (0.5, 1, 2, 4 and 8 μM) in the absence or presence of 0.2 or 0.5 μM NES, NoMAC, DRSP or trilostane. The steroid containing media (500 μl) were removed at specific time intervals and the steroids extracted using a 3:1 volume of tert-Butyl methyl ether (MTBE) to culture medium as previously described [51]. Briefly, the samples were vortexed for 10 minutes, incubated at -80°C for 1–2 hours allowing the medium (aqueous phase) to freeze, whereafter the MTBE phase containing the steroids were transferred to clean test tubes and dried at 50°C under nitrogen. The dried steroid residue was resuspended in 200 μl 50% methanol, vortexed for 2 minutes and stored at -20°C prior to analysis by UPLC–MS/MS. The cells were washed with 1x PBS, lysed with passive lysis buffer (0.2% (v/v) Triton, 10% (v/v) glycerol, 2.8% (v/v) TRIS-phosphate-EDTA and 1.44 mM EDTA) and the total protein concentration determined using the Bradford protein assay method [49]. The NonlinearModelFit function of Mathematica (http://www.wolfram.com) was used to estimate the kinetic parameters for 3βHSD2 activity and the inhibition constants (K_i_) for NES, NoMAC, DRSP and trilostane.

### Separation and quantification of steroid metabolites and progestins using UPLC-MS/MS

Steroid metabolites and progestins were separated using a high strength silica (HSS) T3 column (2.1 mm × 50 mm, 1.8 μm) coupled to an ACQUITY UPLC (Waters, Milford, USA) as previously described [52]. The mobile phases consisted of (A) 1% formic acid and (B) 100% methanol. The injection volume of each sample was 5 μl and the steroid metabolites and progestins were eluted at a flow rate of 0.600 ml per minute using a linear gradient from 55% A to 75% B in 5 minutes. For the kinetic analysis, Preg and Prog were separated using a linear gradient from 40% A to 80% B in 1.5 min. A Xevo TQ or Xevo TQ-S triple quadrupole mass spectrometer (Waters, Milford, USA) was used in multiple reaction monitoring (MRM) mode using an electrospray in the positive ionization mode (ESI+). The following settings were used: Capillary voltage of 3.5 kV, cone voltage 15–30 V, collision energy 4–20 eV, source temperature 140°C, desolvation temperature 400°C, desolvation gas 800 L/h and cone gas 50 L/h. The MassLynx version 4 software program was used for data collection and analysis.

### Quantitative real-time PCR (qPCR)

H295R cells were seeded into 12-well plates at 1 x 10^5^ cells per well, and two days later treated with DMSO (vehicle control) or 1 μM NES, NoMAC or DRSP for 6 hours. Total RNA was isolated using Tri-reagent (Sigma-Aldrich, South Africa) according to the manufacturer’s instructions, and subsequently reversed transcribed using ImProm-II Reverse Transcription System cDNA synthesis kit (Promega). Real-time qPCR was performed by using the Roche LightCycler^®^ 96 and KAPA SYBR FAST qPCR master mix. The mRNA expression of steroidogenic enzymes and the reference gene *GAPDH* was measured using the following primer sets: *CYP17A1* [53], 5-TGGCCCCATCTATTCTGTTCG-3’ (forward primer) and 5’-TAGAGTTGCCATTTGAGGCCG-3’ (reverse primer); *3βHSD2* [54], 5’-TGCCAGTCTTCATCTACACCAG-3’ (forward primer) and 5’-TTCCAGAGGCTCTTCTTCGTG -3’ (reverse primer); *GAPDH* [55], 5’-TGAACGGGAAGCTCACTGG-3’ (forward primer) and 5’-TCCACCACCCTGTTGCTGTA-3’. The relative transcript levels of the target genes were calculated using the method described by [56], and normalised to the relative transcript levels of *GAPDH*.

### Data manipulation and statistical analysis

GraphPad Prism^®^ software version 5 was used for data manipulations, graphical presentations and statistical analysis. One-way ANOVA with Dunnett’s (compares all columns versus control column) post-test was used for statistical analysis. Statistically significant differences are indicated by either *, **, *** to indicate p<0.05, p<0.01 or p<0.001, respectively, whereas p>0.05 indicates no statistical significance (ns). The error bars represent the standard error of the mean (SEM) of at least two independent experiments. The kinetic parameters for 3βHSD2 activity and the inhibition constants (K_i_) for NES, NoMAC, DRSP and trilostane were fitted by minimizing the sum of the squared differences between the data sets and the models, using the NonlinearModelFit function of Mathematica (http://www.wolfram.com). Data was fitted to three different inhibition mechanisms: a competitive inhibition mechanism (inhibitor binds only to the free enzyme), a non-competitive inhibition mechanism (inhibitor binds to both the free enzyme and the enzyme-substrate complex) and an uncompetitive inhibition mechanism (inhibitor binds only to the enzyme-substrate complex).

## Results

### NES and NoMAC modulate steroid production by the human H295R adrenocortical carcinoma cell line

**Fig 3:**
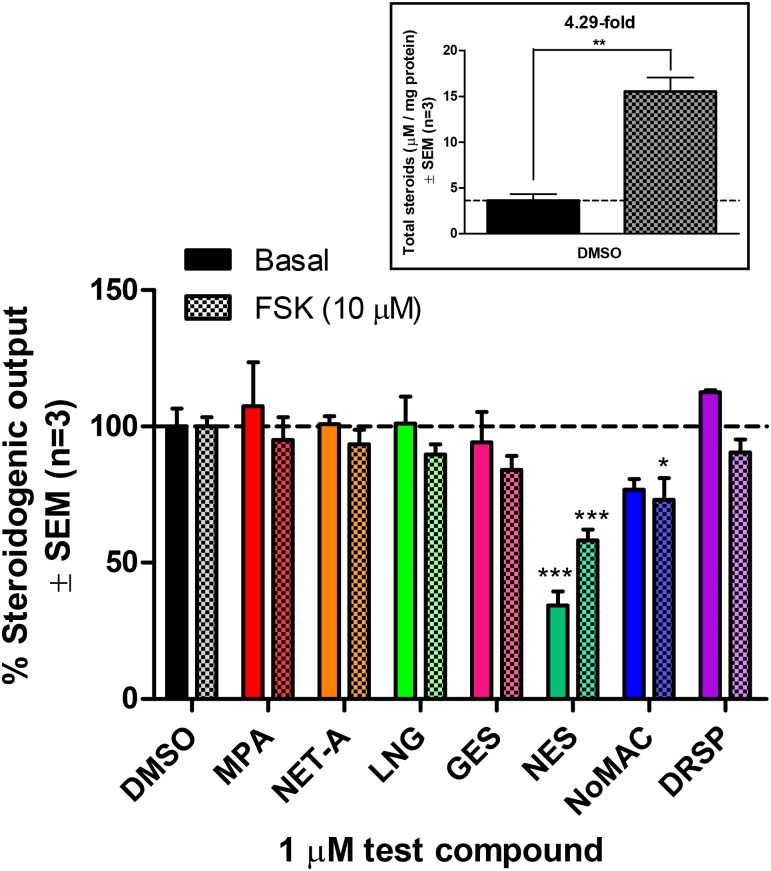
Effect of selected progestins on total steroid production by the human H295R adrenocortical carcinoma cell line under basal and FSK-stimulated conditions. Cells were incubated with DMSO (vehicle control) or 1 μM MPA, NET-A, LNG, GES, NES, NoMAC or DRSP, in the absence and presence of 10 μM FSK for 48 hours. Steroid metabolites were extracted from the cell culture medium and analyzed by UPLC–MS/MS. The concentrations of total steroid produced (μM) were normalized to protein concentration (mg/ml). The insert graph shows the total steroid production (μM/mg protein) in the absence of progestin treatment (DMSO) under basal and FSK-stimulated conditions. This total steroid production for both conditions was set as 100%, and the percentage change upon treatment with progestin relative to the vehicle control (DMSO) of each condition was plotted. Results shown are the average of three independent experiments with each condition performed in triplicate (± SEM).

To assess whether the progestins influence the biosynthesis of endogenous adrenal steroids, the human H295R adrenocortical carcinoma cell line was treated with DMSO or 1 μM MPA, NET-A, LNG, GES, NES, NoMAC or DRSP in the absence and presence of 10 μM forskolin (FSK) for 48 hours, prior to steroid analysis by UPLC–MS/MS. FSK mimics the stimulatory effects of adrenocorticotropic hormone (ACTH) [57] which increases the basal gene expression of endogenous steroidogenic enzymes, resulting in increased steroid production [32, 54]. Indeed, treatment with FSK resulted in a 4.29-fold increase in the total amount of steroids produced by the H295R cells (Fig 3 insert; S1 Table). Interestingly, results in Fig 3 show that total steroid output was not affected by the first- (MPA and NET-A), second- (LNG) or third- (GES) generation progestins, but differentially influenced by the fourth-generation progestins. NES, but not DRSP, significantly inhibited the steroidogenic output by the H295R cells under both basal and FSK-stimulated conditions. Notably, even though NoMAC appeared to inhibit the steroidogenic output under both these conditions, output inhibition under basal conditions was not statistically significant. MTT cell viability assays revealed that the inhibitory effects observed for NES and NoMAC were not due to a decrease in cell viability (S1 Fig).

Upon closer inspection of the effects of the progestins on basal and FSK-stimulated production of steroid intermediates and end products in the steroidogenic pathway (summarised in Tables 1 and 2), it is clear that the fourth-generation progestins, NES, NoMAC and DRSP, modulate the synthesis of numerous endogenous steroids. These progestins appeared to increase the basal and FSK-stimulated concentrations of Preg, the first metabolite in the steroidogenic pathway, while in most cases the concentrations of the Δ^4^ C21 steroids Prog, 17OH-Prog, 16OH-Prog, DOC, and CORT were reduced by NES and NoMAC, but not DRSP (Tables 1 and 2). DRSP increased the basal production of DOC, but had no effect on the production of steroids from the mineralocorticoid pathway in the presence of FSK. However, similarly to NES and NoMAC, DRSP inhibited the basal and FSK-stimulated production of deoxycortisol. Like NES, but unlike NoMAC, DRSP lowered the basal concentration of the glucocorticoid cortisol. Furthermore, NES and NoMAC also tended to decrease the concentrations of the Δ^4^ C19 androgen precursors A4 and 11OH-A4 as well as the Δ^4^ C19 androgen testosterone. Although both NES and NoMAC increased the concentration of the Δ^5^ C19 adrenal androgen precursor DHEA under basal conditions, this increase was not significant in the case of NoMAC. Lastly, DRSP displayed similar inhibitory effects to that of NES and NoMAC on the production of the Δ^4^ C19 androgen precursors and androgens. Interestingly, progestins from the first three generations had no effect on the synthesis of end products under both basal and FSK-stimulated conditions (Tables 1 and 2), but modulated the synthesis of some intermediates in the presence of FSK (Table 2). For example, MPA increased the synthesis of the androgen precursors, A4 and 11OH-A4, while GES and LNG inhibited A4 and 11-DHC, respectively.

**Table 1:** Fold change in basal steroid production in response to selected progestins^a^.

Steroid metabolite	MPA (1^st^)	NET-A (1^st^)	LNG (2^nd^)	GES (3^rd^)	NES (4^th^)	NoMAC (4^th^)	DRSP (4^th^)
Preg	-	-	-	-	↑ 2.87 ± 0.57 ***	↑ 2.17 ± 0.19 **	↑ 4.02 ± 0.33 ***
Prog	-	-	-	-	↓ 30.30 ± 0.02 ***	↓ 2.34 ± 0.12 *	↑ 11.11 ± 2.24 ***
17OH-Prog	-	-	-	-	↓ 8.33 ± 0.00 ***	-	↑ 6.32 ± 0.51 ***
16OH-Prog	-	-	-	-	↓ 14.18 ± 0.02 ***	↓ 3.41 ± 0.03 **	↑ 2.50 ± 0.68 *
DOC	-	-	-	-	↓ 7.68 ± 0.08 **	↓ 1.73 ± 0.07 ^ns^	↑ 2.85 ± 0.30 ***
CORT	-	-	-	-	↓ 2.73 ± 0.26 *	↓ 1.43 ± 0.13 ^ns^	↑ 1.58 ± 0.04 ^ns^
11-DHC	-	-	-	-	↑ 2.53 ± 1.42 ^ns^	↑ 1.49 ± 0.11 ^ns^	↑ 2.07 ± 0.52 ^ns^
Ald	-	-	-	-	-	-	-
Deoxycortisol	-	-	-	-	↓ 6.12 ± 0.01 ***	↓ 1.43 ± 0.07 **	↓ 1.81 ± 0.08 ***
Cortisol	-	-	-	-	↓ 2.97 ± 0.16 **	-	↓ 2.80 ± 0.05 **
Cortisone	-	-	-	-	↑ 5.33 ± 1.11 ***	-	↓ 1.82 ± 0.17 ^ns^
DHEA	-	-	-	-	↑ 26.54 ± 5.50 ***	↑ 1.70 ± 0.30 ^ns^	↑ 1.85 ± 0.08 ^ns^
A4	-	-	-	-	↓ 7.12 ± 0.03 ***	-	↓ 1.75 ± 0.14 ^ns^
11OH-A4	-	-	-	-	-	-	↓ 3.19 ± 0.19 *
Testosterone	-	-	-	-	↓ 3.77 ± 0.17 ***	↓ 1.58 ± 0.03 *	↓ 1.79 ± 0.04 *
**Total steroid (μM)**	**-**	**-**	**-**	**-**	**↓ 2.91 ± 0.05** **	**-**	**-**

^a^The human H295R cell line was treated with DMSO (vehicle control) or 1 μM MPA, NET-A, LNG, GES, NES, NoMAC or DRSP for 48 hours.

Steroids were extracted and quantified by UPLC–MS/MS. The fold change ± SEM in response to progestin treatment relative to the vehicle control (DMSO), which was set as one, is indicated. (-) denotes no effect; 17OH-Preg, DHT, estrone and 17β-estradiol were below the limit of detection in the control samples and thus fold changes in the levels of these steroids in the presence of progestins could not be determined.

Statistically significant differences are indicated by either *, **, *** to indicate p<0.05, p<0.01 or p<0.001, respectively.

**Table 2:** Fold change in FSK-stimulated steroid production in response to selected progestins^b^.

Steroid metabolite	MPA (1^st^)	NET-A (1^st^)	LNG (2^nd^)	GES (3^rd^)	NES (4^th^)	NoMAC (4^th^)	DRSP (4^th^)
Preg	-	-	-	-	↑ 3.35 ± 0.35 ***	↑ 1.47 ± 0.06 ^ns^	↑ 1.28 ± 0.11 ^ns^
Prog	-	-	-	-	↓ 12.55 ± 0.03 ***	↓ 1.63 ± 0.08 *	↑ 3.16 ± 0.74 ***
17OH-Prog	-	-	-	-	↓ 3.64 ± 0.03 **	-	↑ 2.35 ± 0.23 ***
16OH-Prog	-	-	-	-	↓ 35.71 ± 0.01 ***	↓ 2.28 ± 0.03 *	-
DOC	-	-	-	-	↓ 34.88 ± 0.01 ***	↓ 3.02 ± 0.02 ***	-
CORT	-	-	-	-	↓ 6.25 ± 0.03 ***	↓ 1.59 ± 0.12 ^ns^	-
11-DHC	-	-	↓ 1.83 ± 0.13 *	-	-	-	-
Ald	-	-	-	-	↓ 2.22 ± 0.19 ^ns^	-	-
Deoxycortisol	-	-	-	-	↓ 10.98 ± 0.02 ***	↓ 1.78 ± 0.03 *	↓ 1.44 ± 0.03 *
Cortisol	-	-	-	-	↓ 1.71 ± 0.16 ^ns^	-	↓ 2.22 ± 0.17 ^ns^
Cortisone	-	-	-	-	↑ 2.98 ± 0.93 *	↑ 2.08 ± 0.35 ^ns^	↓ 1.73 ± 0.11 ^ns^
DHEA	-	-	-	-	-	-	↓ 2.19 ± 0.13 *
A4	↑ 1.29 ± 0.08 **	-	-	↓ 1.38 ± 0.00 **	↓ 13.33 ± 0.03 ***	↓ 1.43 ± 0.06 **	↓ 1.92 ± 0.05 ***
11OH-A4	↑ 1.61 ± 0.00 ***	-	-	-	↓ 2.43 ± 0.03 ***	↑ 1.77 ± 0.10 ***	↓ 2.34± 0.04 ***
Testosterone	-	-	-	-	↓ 18.29 ± 0.04 ***	↓ 1.49 ± 0.02 *	↓ 1.65 ± 0.14 *
**Total steroid (μM)**	**-**	**-**	**-**	**-**	**↓ 1.72 ± 0.04** **	**↓ 1.37 ± 0.08** *	**-**

^b^The human H295R cell line was treated with DMSO (vehicle control) or 1 μM MPA, NET-A, LNG, GES, NES, NoMAC or DRSP in the presence of FSK for 48 hours.

Steroids were extracted and quantified by UPLC–MS/MS. The fold change ± SEM in response to progestin treatment relative to the vehicle control (DMSO), which was set as one, is indicated. (-) denotes no effect; 17OH-Preg, DHT, estrone and 17β-estradiol were below the limit of detection in the control samples and thus fold changes in the levels of these steroids in the presence of progestins could not be determined.

Statistically significant differences are indicated by either *, **, *** to indicate p<0.05, p<0.01 or p<0.001, respectively.

**Fig 4:**
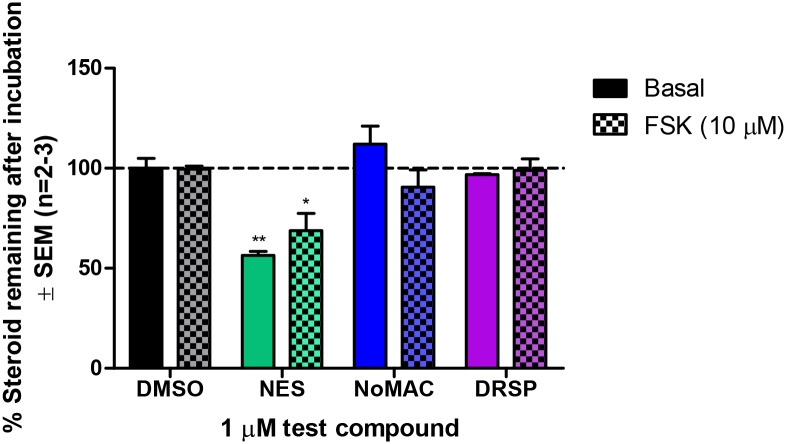
NES is metabolized by the H295R cells under both basal and FSK-stimulated conditions. H295R cells were treated with DMSO or 1 μM NES, NoMAC or DRSP in the absence and presence of 10 μM FSK for 48 hours. Medium containing the test compounds (no cells) was added to the wells of a 12-well plate as a negative control for metabolism. Steroids were extracted and analyzed by UPLC–MS/MS. The amount of progestin present in the medium after incubation with the cells was expressed as a % relative to the amount of progestin in the negative control for metabolism, which was set as 100%. Result shown is the average of at least two independent experiments with each condition performed in triplicate (± SEM).

We subsequently investigated whether NES, NoMAC and DRSP are metabolized in the H295R cell line and observed a significant reduction in the concentration of NES after the 48 hour incubation period suggesting that this progestin is metabolized by the cells. It should however be noted that more than 50% of this progestin was still unmetabolized after the incubation period. Conversely, NoMAC and DRSP were not metabolized (Fig 4). This result suggests that the observed effects in the presence of NoMAC and DRSP on steroid biosynthesis are due to the progestins themselves, while the effects observed for NES may be attibuted to NES itself and/or its metabolites.

### NES and NoMAC inhibit the activity of 3βHSD2, while 3βHSD2 and CYP17A1 activities are inhibited by DRSP

**Fig 5:**
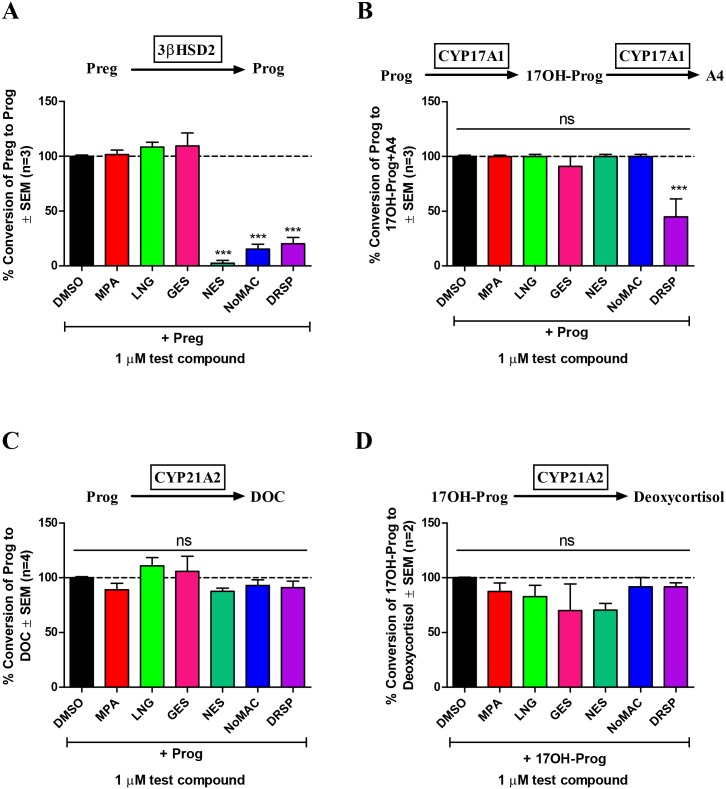
NES, NoMAC and DRSP inhibit the activity of human 3βHSD2, while only DRSP inhibits CYP17A1 activity. COS-1 cells were transiently transfected with plasmids expressing human (A) 3βHSD2 (pCDNA6-hHSD3β2-V5), (B) CYP17A1 (pIRES-hCYP17A1-V5-X-hCYPB5-6HIS) or (C and D) CYP21A2 (pCDNA6-hCYP21A2-V5), respectively. Cells were subsequently treated with 1 μM Preg (A) or Prog (B and C) or 17OH-Prog (D), in the absence (DMSO) and presence of 1 μM MPA, LNG, GES, NES, NoMAC or DRSP for 20 minutes (A), 4 hours (B) or 90 minutes (C and D), respectively. The steroid metabolites produced by the cells in the medium were extracted and analyzed by UPLC–MS/MS. The concentration of the steroids produced by the cells was normalized to the total protein concentration using the Bradford protein assay method. The % conversion of substrate to product was plotted, with the substrate only response (DMSO) set as 100% and everything else relative to that. Results shown are the average of at least two independent experiments with each condition performed in triplicate (± SEM).

Due to the modulation of steroidogenesis by NES, NoMAC and DRSP observed in the H295R cell line we next determined whether the activity of specific steroidogenic enzymes could be influenced by these three progestins. The increased production of Δ^5^ steroids coupled to the decrease in Δ^4^ steroid concentrations observed in most cases, suggested that the progestins may be modulating the activity and/or expression of 3βHSD2. We also investigated the ability of the progestins to modulate the activity of CYP17A1 and cytochrome P450 21-hydroxylase (CYP21A2), for which the natural progestogen, Prog, is a substrate. Non-steroidogenic COS-1 cells were transiently transfected with the cDNA expression vectors for the human 3βHSD2, CYP17A1 and CYP21A2 enzymes, respectively, followed by treatment with the appropriate steroid substrate in the absence (DMSO) or presence of 1 μM MPA, LNG, GES, NES, NoMAC or DRSP. In addition to the fourth-generation progestins, one progestin from the earlier generations was included. Effects on the activity of 3βHSD2 was assessed using Preg as substrate (Fig 5A), while Prog was used as substrate to examine the effects on the activity of CYP17A1 (Fig 5B). Prog and 17OH-Prog were both used as substrates for investigating the effects on the activity of CYP21A2 (Fig 5C and 5D). As shown in Fig 5A, NES (97.44 ± 2.56%), NoMAC (84.52 ± 4.35%) and DRSP (79.77 ± 5.69%) significantly inhibited the activity of 3βHSD2. Interestingly, DRSP was the only progestin that inhibited the activity of CYP17A1 (55.20 ± 16.50%), while none of the progestins inhibited CYP21A2 activity (Fig 5C and 5D). None of the first-, second- or third-generation progestins affected the activity of the above-mentioned enzymes.

**Fig 6:**
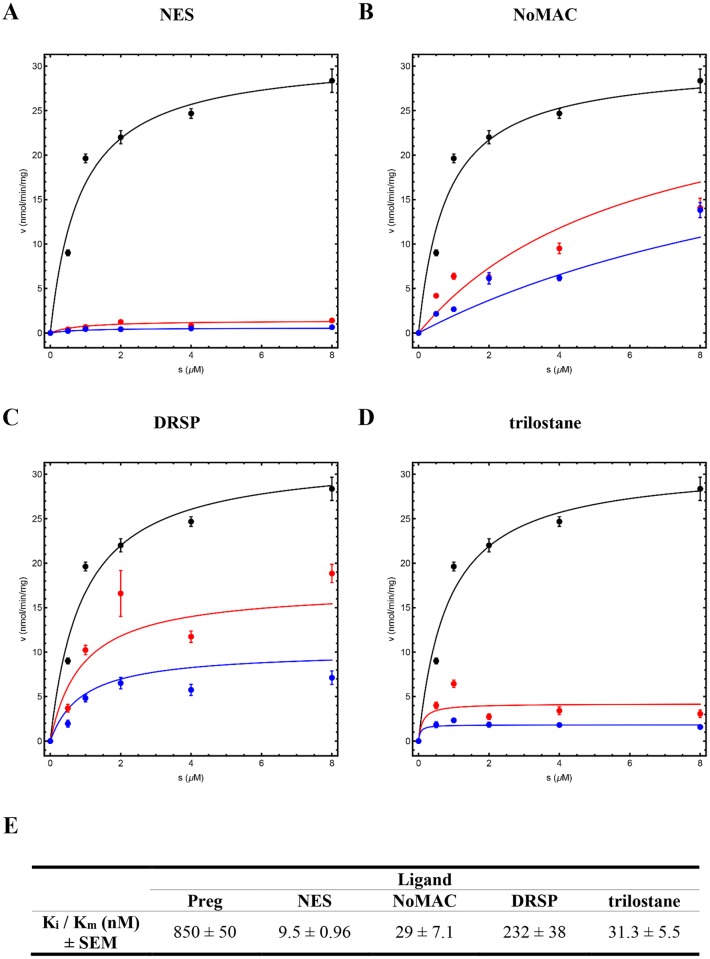
Inhibition of 3βHSD2 activity by NES, NoMAC, DRSP and trilostane. COS-1 cells were transiently transfected with a plasmid expressing human 3βHSD2 (pCDNA6-hHSD3β2-V5), and subsequently treated with increasing concentrations (0.5, 1, 2, 4 and 8 μM) of Preg (substrate) in the presence of 0.0, 0.2 or 0.5 μM (A) NES, (B) NoMAC, (C) DRSP or (D) trilostane. The conversion of Preg to Prog was analyzed using UPLC-MS/MS. Michaelis-Menten plots are shown in the absence (black symbols and lines) and presence of 0.2 μM (red symbols and lines) and 0.5 μM (blue symbols and lines) of NES, NoMAC, DRSP and trilostane. A K_m_ of 0.85 ± 0.05 μM and V_max_ of 31.1 ± 0.7 nmol/min/mg were obtained in the absence of inhibitor. Three inhihitory mechanisms were fitted (S2 Fig) and the best fit mechanism is shown. Each data point represents the mean ± SE of one experiment performed in duplicate. These results were validated by a model predicting 3βHSD2 activity in an independent experiment (S3 Fig, dashed green line).

Having shown that NES, NoMAC and DRSP abrogate the ability of 3βHSD2 to convert Preg to Prog (Fig 5A), we next determined the K_i_ values of these inhibitors as well as that of the well-known 3βHSD inhibitor trilostane, serving as a positive control [58]. COS-1 cells were transiently transfected with the cDNA expression vector for the human 3βHSD2, followed by treatment with Preg in the absence or presence NES, NoMAC, DRSP or trilostane. In the absence of inhibitor, a K_m_ of 0.85 ± 0.05 μM and V_max_ of 31.1 ± 0.7 nmol/min/mg were obtained. The fits for all inhibitory mechanisms are shown in S2 Fig, while the results in Fig 6 show the fits with the mechanisms best describing the data. These fits resulted in a K_i_ value of 9.5 ± 0.96 nM for NES (with a non-competitive mechanism), 29 ± 7.1 nM for NoMAC (with a competitive mechanism), 232 ± 38 nM for DRSP (with a non-competitive mechanism) and 31.3 ± 5.5 nM for trilostane (with an uncompetitive mechanism). We subsequently used these K_i_ values to predict the 3βHSD2 activity when 1 μM of the inhibitor and 1 μM of the substrate are used (S3 Fig, dashed green line). We show that the residual activities predicted for NES (0.16 ± 0.03 nmol/min/mg), NoMAC (1.04 ± 0.30 nmol/min/mg) and DRSP (3.16 ± 0.87 nmol/min/mg) correlate with the experimental data (NES, 0.43 ± 0.01 nmol/min/mg; NoMAC, 2.62 ± 0.11 nmol/min/mg; DRSP, 3.39 ± 0.19 nmol/min/mg) obtained from Fig 5A, thus validating the determined K_i_ values.

**Fig 7:**
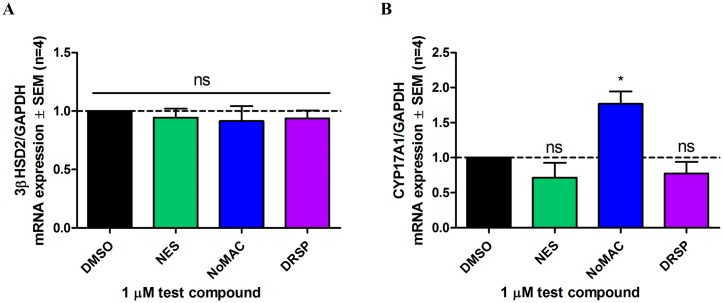
NES, NoMAC and DRSP do not inhibit the mRNA expression of *3βHSD2* and *CYP17A1* in the human H295R adrenocortical carcinoma cell line. The H295R cell line was incubated with DMSO (vehicle control) or 1 μM NES, NoMAC or DRSP for 6 hours. Total RNA was isolated, reversed transcribed to cDNA and real-time qPCR performed to determine the relative mRNA expression levels of (A) *3βHSD2* and (B) *CYP17A1*. *GAPDH* was used as the reference gene. Results shown are the average of four independent experiments with each condition performed in duplicate (± SEM).

Despite our observation that NES, NoMAC and DRSP inhibit the activity of 3βHSD2, and that DRSP also inhibits the activity of CYP17A1, it is possible that the modulation of steroidogenesis seen in H295R cells could also be due to the progestins altering the expression levels of these enzymes. We therefore used real-time qPCR to investigate the effect of NES, NoMAC and DRSP on the mRNA levels of *3βHSD2* and *CYP17A1* in H295R cells. The cells were treated with DMSO (vehicle control) or 1 μM NES, NoMAC or DRSP for 6 hours, followed by real-time qPCR analysis for the expression of *3βHSD2* and *CYP17A1*, respectively. The results in Fig 7A shows that none of the fourth-generation progestins inhibited the mRNA expression of the *3βHSD2* gene. Furthermore, our results show that neither NES nor DRSP inhibited the mRNA expression of *CYP17A1* (Fig 7B). Surprisingly, we show that NoMAC upregulated the mRNA expression of the *CYP17A1* gene.

## Discussion

Progestins are classified into four generations and are widely used in endocrine therapies by pre- and post-menopausal women. To date, only a few studies have investigated the effects of these compounds on the biosynthesis of endogenous steroids, and studies directly comparing the effects of different progestins in the same model system are lacking. To the best of our knowledge, the present study is the first to directly compare the effects of select progestins from all four generations on the production of both intermediates and end products of the steroidogenic pathway (Fig 1) in the human H295R adrenocortical carcinoma cell line. Surprisingly, although earlier studies have shown that progestins from the first-, second- and fourth-generation reduce the production of some endogenous steroids, we show that only the fourth-generation progestins NES, NoMAC and DRSP modulate the biosynthesis of endogenous steroids in H295R cells. Cell viability assays showed that these changes were not due to changes in cell viability (S1 Fig). The general trend observed was that these progestins decreased the concentrations of steroids in the glucocorticoid and androgen pathways, while the production of steroids in the progestogen and mineralocorticoid pathways were decreased by NES and NoMAC, and increased by DRSP. The observation that NES and NoMAC elicited mostly similar effects on steroidogenesis, but different to that of DRSP, may be due to the fact that NES and NoMAC are structurally similar (reviewed in [3]), while DRSP has a unique structure derived from the MR antagonist spironolactone [59, 60]. Furthermore, we found that NES, but not NoMAC and DRSP, is metabolized in the H295R cells (Fig 4), suggesting that the effects of NoMAC and DRSP are due to the progestins themselves, while the effect of NES may be due to NES itself, its metabolites or a combination thereof. The identification of the metabolites in the H295R cells was however, beyond the scope of the current study.

To understand the mechanism whereby the fourth-generation progestins modulate adrenal steroid biosynthesis, we investigated the effects of the progestins on the activity and/or expression of 3βHSD2, CYP17A1 and CYP21A2. 3βHSD2 was investigated as NES and NoMAC tended to increase the concentrations of the Δ^5^ C21 steroid Preg (Tables 1 and 2) and the Δ^5^ C19 steroid DHEA (Table 1), while the production of several Δ^4^ C21 (Prog, 17OH-Prog, 16OH-Prog, DOC, CORT, deoxycortisol and cortisol) and Δ^4^ C19 (A4, 11OH-A4 and testosterone) steroids were decreased. DRSP also increased the production of Preg and decreased the production of A4, 11OH-A4 and testosterone (Δ^4^ C19 steroids), while differentially affecting the production of Δ^4^ C21 steroids, suggesting inhibition of additional steroidogenic enzymes. Moreover, as progestins were designed to mimic Prog, and considering that Prog is a substrate for both CYP17A1 and CYP21A2, the possibility that progestins modulate the activities and/or expression of these enzymes could not be excluded.

In COS-1 cells transfected to constitutively express human 3βHSD2 (Fig 5A), CYP17A1 (Fig 5B) and CYP21A2 (Fig 5C and 5D), respectively, we show that NES and NoMAC had no effect on the activity of CYP17A1 or CYP21A2, but that these progestins significantly inhibited the activity of 3βHSD2. DRSP also had no effect on the activity of CYP21A2 (Fig 5C and 5D), but inhibited the activities of both 3βHSD2 (Fig 5A) and CYP17A1 (Fig 5B). The inhibition of 3βHSD2 in COS-1 cells by DRSP correlates with the observed increase in the concentration of Preg and decrease in the concentrations of the Δ^4^ C19 steroids observed in the H295R cells, while the accumulation of Prog, 16OH-Prog and 17OH-Prog in the H295R cells is likely due to a bottleneck caused by the simultaneous inhibition of 3βHSD2 and CYP17A1. Discrepancies between the inhibition observed in COS-1 cells and the results observed in the H295R cells may further be explained by the once-off addition of substrate in the case of the assays performed in COS-1 cells, which is in contrast to the H295R cells which continuously produce steroids and also express multiple enzymes which may compete for binding to the same substrate.

Notably, our real-time qPCR results show that neither NES, NoMAC nor DRSP inhibit the mRNA expression of *3βHSD2* in H295R cells (Fig 7A), and that DRSP has no significant effect on *CYP17A1* gene expression (Fig 7B). It was interesting to note that although *CYP17A1* mRNA expression was increased in the presence of NoMAC, this did not translate to an observed increase in activity. While the possibility that NES, NoMAC and DRSP modulate the protein levels of these steroidogenic enzymes cannot be excluded, our COS-1 data confirms enzyme inhibition of 3βHSD2. Subsequent kinetic studies suggest that the K_i_ values determined for these progestins are similar to that of the well-known 3βHSD2 inhibitor trilostane. Although the mechanism of inhibition that best fitted the data (Fig 6) suggest that trilostane, unlike NES, NoMAC and DRSP, is an uncompetitive inhibitor of 3βHSD2, it should be noted that a similar fit was also obtained with the non-competitive mechanism (S2 Fig). Trilostane has previously been reported to inhibit the activity of 3βHSD2 via a non-competitive mechanism [61]. The fitted data suggest that NES and DRSP are non-competitive inhibitors of 3βHSD2, while NoMAC is a competitive inhibitor (Fig 6). It is noteworthy that the K_i_ values determined for the fourth-generation progestins in this study were validated by their ability to independently predict the inhibition of 3βHSD2 activity in the presence of 1 μM substrate and inhibitor (S3 Fig).

To our knowledge, our study is the first to show that NES and/or its metabolites, NoMAC and DRSP differentially suppress adrenal steroid biosynthesis and that this inhibition in the production of steroid hormones in the H295R cells are in line with the inhibition of human 3βHSD2 activity in the COS-1 cells. Despite the fact that other studies did not investigate the effects of these fourth-generation progestins on the activity and/or mRNA expression of 3βHSD2, effects have been reported for first- and second-generation progestins [34, 37–39]. Our results are in agreement with the findings that MPA [37], as well as NET and LNG [38] have no effects on the activity of rat ovarian 3βHSD. Conversely, using a yeast expression system, Lee et al. have previously shown an inhibition of 3βHSD2 by MPA and determined a K_i_ of 3 μM [34]. Despite this relatively high K_i_ treatment of breast cancer patients with high doses of MPA (serum concentrations of 0.14–1.7 μM) have previously been shown to decrease the serum levels of cortisol, A4, DHEA-S and testosterone [24–27, 62, 63]. While we did not observe inhibition with 1 μM MPA in our test system, we show potent inhibition of 3βHSD2 by NES, NoMAC and DRSP. Considering their potent K_i_ values, which are in the nanomolar range and an order of magnitude lower than the K_i_ determined for MPA by Lee and co-workers (1999), it is likely that NES, NoMAC and DRSP modulate steroid levels *in vivo*. Furthermore, it is important to note that these validated K_i_ values fall within the serum ranges reported for the contraceptive usage of NES (0.086–27.3 nM), NoMAC (3–33 nM) and DRSP (26.7–253 nM), further highlighting the potential of these progestins to modulate steroid levels *in vivo* [64–71]. Indeed, results showing decreased concentrations of mineralocorticoids and glucocorticoids in the presence of NES and NoMAC, likely by the inhibition of 3βHSD2, suggest that the use of these fourth-generation progestins may be beneficial for women suffering from metabolic syndromes and/or CVDs caused by glucocorticoid and mineralocorticoid excess [21, 68–70].

Furthermore, it has previously been shown that when DRSP was combined with ethinyl estradiol in a combined oral contraceptive and administered to hyperandrogenic women diagnosed with polycystic ovary syndrome (PCOS), serum concentrations of total and free testosterone, A4 and DHEA-S were decreased [28]. Although the authors did not investigate the effect of DRSP on the activity of any steroidogenic enzyme, they suggested that the decrease may be due to inhibition of the 17α-hydroxylase and 17,20-lyase activities of CYP17A1, as they found a decease in the ratio of 17OH-Prog/Prog and A4/17OH-Prog. Our study in the COS-1 cells directly investigating the inhibition of CYP17A1, showed that DRSP does indeed inhibit the activty of this enzyme, and also the activity of 3βHSD2. While previous studies showed that MPA inhibits the 17α-hydroxylase activity of rat ovarian CYP17A1 [37], our results are in agreement with others showing that MPA has no effect on the activity of the human CYP17A1 [34]. Taken together, the inhibition of androgen production observed with the fourth-generation progestins in our study, but not earlier generation progestins like MPA, suggest that the use of the fourth-generation progestins may have better therapeutic benefits for women with hyperandrogenism associated disorders such as PCOS than the earlier generations. Although a number of different progestins are used in the treatment of PCOS, the degree of androgenicity of the progestin is an important consideration. The fact that NES, NoMAC and DRSP do not display any androgenic properties, while the selected earlier generation progestins used in this study do [3], further supports the preferential use of the fourth-generation progestins to treat PCOS.

The implications of decreased androgen production in other disorders or diseases such as breast cancer, however, are not straightforward. For example, as epidemiological and case-control studies indicate an association between elevated concentrations of androgens and increased risk of developing breast cancer [72–75], decreased androgen production may be advantageous in terms of androgen receptor (AR)-positive breast cancers. Conversely, the observed decrease of androgens may be detrimental as androgens and the AR have been proposed to have protective roles in breast cancer (reviewed in [76, 77]). This complexity is further highlighted by the fact that the use of both an androgenic progestin (MPA) and a non-androgenic progestin (NoMAC) used in HRT were shown to be associated with an increased risk of developing breast cancer in postmenopausal women [78].

## Conclusion

In summary, all three of the fourth-generation progestins investigated in this study had effects on steroidogenesis, with effects observed with NES and NoMAC being mostly similar, while those observed for DRSP often differed. The results showing that NES, NoMAC and DRSP inhibit 3βHSD2 activity, while DRSP inhibits the activities of both 3βHSD2 and CYP17A1 in the COS-1 cells, correlate to the changes observed in the biosynthesis of steroid hormones in the H295R cell line. Although the concentration (1 μM) of the progestins used in this study are supraphysiological, the K_i_ values determined for the inhibition of 3βHSD2 fall within the serum ranges reported for the contraceptive usage of NES, NoMAC and DRSP, supporting the likelihood that these progestins affect adrenal steroidogenesis *in vivo*. The findings of our study further highlight the fact that, although progestins are all designed to mimic the biological activity of Prog, relatively minor differences in their structures may cause profound alterations in their biochemical activity.

## Supporting Information

S1 Fig Viability of basal and forskolin (FSK)-stimulated H295R cells in the presence of different generation progestins. Cells were incubated for 48 hours with DMSO (vehicle control) or 1 μM MPA, NET-A, LNG, GES, NES, NoMAC or DRSP in the absence or presence of 10 μM FSK. Cell viability was measured using the MTT assay and results are expressed as fold proliferation relative to DMSO = 1. Results shown are the average of three independent experiments (±SEM) performed in triplicate.
(TIF)

S2 Fig Michaelis-Menten plots of 3βHSD activity in the absence or presence of NES, NoMAC, DRSP and trilostane. COS-1 cells were transiently transfected with a plasmid expressing human 3βHSD2 (pCDNA6-hHSD3β2-V5), and subsequently treated with Preg (0.5, 1, 2, 4 and 8 μM) in the presence of 0.0, 0.2 or 0.5 μM NES, NoMAC, DRSP or trilostane. The conversion of Preg to Prog was analyzed using UPLC-MS/MS. Three inhibitory mechanisms were fitted to the data sets: competitive, non-competitive and uncompetitive, using the rate equations shown in the figure. Confidence intervals (95%) for the fits are indicated in the plots with grey fillings. Each data point represents the mean ± SE of at least duplicate experiments.
(TIF)

S3 Fig Predicted Michaelis-Menten plots of 3βHSD2 in the presence of 1 μM NES, NoMAC and DRSP. Michaelis-Menten plots were predicted (dashed green line) based on the data presented in Fig 6. The predicted V_max_ in the presence of 1 μM NES (0.16 ± 0.03 nmol/min/mg), NoMAC (1.04 ± 0.30 nmol/min/mg) and DRSP (3.16 ± 0.87 nmol/min/mg) correlates with the residual activities determined experimentally (NES, 0.43 ± 0.01 nmol/min/mg; NoMAC, 2.62 ± 0.11 nmol/min/mg; DRSP, 3.39 ± 0.19 nmol/min/mg) as shown in Fig 5A.
(TIF)

S1 Table Basal and FSK-stimulated production of steroid metabolites in the human adrenal H295R cell line. (PDF)

## References

[1] Speroff L, A Clinical Guide for Contraception. 2nd ed 1996: Baltimore: Williams & Wilkins, Baltimore, MD.

[2] Hapgood JP, Africander D, Louw R, Ray RM, Rohwer JM. Potency of progestogens used in hormonal therapy: toward understanding differential actions. J Steroid Biochem Mol Biol. 2014; 142: 39–47. 10.1016/j.jsbmb.2013.08.001 23954501

[3] Stanczyk FZ, Hapgood JP, Winer S, Mishell DR Jr. Progestogens Used in Postmenopausal Hormone Therapy: Differences in Their Pharmacological Properties, Intracellular Actions, and Clinical Effects. Endocr Rev. 2013; 34(2). 10.1210/er.2012-1008 23238854 PMC3610676

[4] Stanczyk FZ. Pharmacokinetics and potency of progestins used for hormone replacement therapy and contraception. Rev Endocr Metab Disord. 2002; 3(3): 211–24. 12215716 10.1023/a:1020072325818

[5] Sitruk-Ware R. New progestogens: a review of their effects in perimenopausal and postmenopausal women. Drugs Aging. 2004; 21(13): 865–83. 10.2165/00002512-200421130-00004 15493951

[6] Africander D, Verhoog N, Hapgood JP. Molecular mechanisms of steroid receptor-mediated actions by synthetic progestins used in HRT and contraception. Steroids. 2011; 76(7): 636–52. 10.1016/j.steroids.2011.03.001 21414337

[7] Schindler AE, Campagnoli C, Druckmann R, Huber J, Pasqualini JR, Schweppe KW, Thijssen JH. Classification and pharmacology of progestins. Maturitas. 2003; 46 Suppl 1: S7–S16. 10.1016/j.maturitas.2003.09.014 14670641

[8] Sitruk-Ware R. Pharmacological profile of progestins. Maturitas. 2008; 61(1–2): 151–7. 10.1016/j.maturitas.2004.01.001 19434887

[9] Koubovec D, Ronacher K, Stubsrud E, Louw A, Hapgood JP. Synthetic progestins used in HRT have different glucocorticoid agonist properties. Mol Cell Endocrinol. 2005; 242(1–2): 23–32. 10.1016/j.mce.2005.07.001 16125839

[10] Africander D, Louw R, Verhoog N, Noeth D, Hapgood JP. Differential regulation of endogenous pro-inflammatory cytokine genes by medroxyprogesterone acetate and norethisterone acetate in cell lines of the female genital tract. Contraception. 2011; 84(4): 423–35. 10.1016/j.contraception.2011.06.006 21920200

[11] Africander D, Louw R, Hapgood JP. Investigating the anti-mineralocorticoid properties of synthetic progestins used in hormone therapy. Biochem Biophys Res Commun. 2013; 433(3): 305–10. 10.1016/j.bbrc.2013.02.086 23473756

[12] Africander DJ, Storbeck KH, Hapgood JP. A comparative study of the androgenic properties of progesterone and the progestins, medroxyprogesterone acetate (MPA) and norethisterone acetate (NET-A). J Steroid Biochem Mol Biol. 2014; 143: 404–15. 10.1016/j.jsbmb.2014.05.007 24861265

[13] Louw-du Toit R, Hapgood JP, Africander D. Medroxyprogesterone acetate differentially regulates interleukin (IL)-12 and IL-10 in a human ectocervical epithelial cell line in a glucocorticoid receptor (GR)-dependent manner. J Biol Chem. 2014; 289(45): 31136–49. 10.1074/jbc.M114.587311 25202013 PMC4223317

[14] Sitruk-Ware R. New progestagens for contraceptive use. Hum Reprod Update. 2006; 12(2): 169–78. 10.1093/humupd/dmi046 16291771

[15] Gronich N, Lavi I, Rennert G. Higher risk of venous thrombosis associated with drospirenone-containing oral contraceptives: a population-based cohort study. CMAJ. 2011; 183(18): E1319–25. 10.1503/cmaj.110463 22065352 PMC3255137

[16] Parkin L, Sharples K, Hernandez RK, Jick SS. Risk of venous thromboembolism in users of oral contraceptives containing drospirenone or levonorgestrel: nested case-control study based on UK General Practice Research Database. BMJ. 2011; 342: d2139 10.1136/bmj.d2139 21511804 PMC3081041

[17] Wu CQ, Grandi SM, Filion KB, Abenhaim HA, Joseph L, Eisenberg MJ. Drospirenone-containing oral contraceptive pills and the risk of venous and arterial thrombosis: a systematic review. BJOG. 2013; 120(7): 801–10. 10.1111/1471-0528.12210 23530659

[18] Chrousos GP, Gold PW. A healthy body in a healthy mind—and vice versa—the damaging power of "uncontrollable" stress. J Clin Endocrinol Metab. 1998; 83(6): 1842–5. 10.1210/jcem.83.6.4908 9626106

[19] VanItallie TB. Stress: a risk factor for serious illness. Metabolism. 2002; 51(6 Suppl 1): 40–5. 10.1053/meta.2002.33191 12040540

[20] Tomlinson JW, Stewart PM. Mechanisms of disease: Selective inhibition of 11beta-hydroxysteroid dehydrogenase type 1 as a novel treatment for the metabolic syndrome. Nat Clin Pract Endocrinol Metab. 2005; 1(2): 92–9. 10.1038/ncpendmet0023 16929377

[21] Vinson GP. Angiotensin II, corticosteroids, type II diabetes and the metabolic syndrome. Med Hypotheses. 2007; 68(6): 1200–7. 10.1016/j.mehy.2006.09.065 17134848

[22] Miller WL, Auchus RJ. The molecular biology, biochemistry, and physiology of human steroidogenesis and its disorders. Endocr Rev. 2011; 32(1): 81–151. 10.1210/er.2010-0013 21051590 PMC3365799

[23] Jones JR, DelRosario L, Soriero AA. Adrenal function in patients receiving medroxyprogesterone acetate. Contraception. 1974; 10(1): 1–12. 10.1016/0010-7824(74)90127-9 4374337

[24] Hellman L, Yoshida K, Zumoff B, Levin J, Kream J, Fukushima DK. The effect of medroxyprogesterone acetate on the pituitary-adrenal axis. J Clin Endocrinol Metab. 1976; 42(5): 912–7. 10.1210/jcem-42-5-912 178684

[25] van Veelen H, Willemse PH, Sleijfer DT, Pratt JJ, Sluiter WJ, Doorenbos H. Adrenal suppression by oral high-dose medroxyprogesterone acetate in breast cancer patients. Cancer Chemother Pharmacol. 1984; 12(2): 83–6. 10.1007/BF00254594 6321047

[26] Lang I, Zielinski CC, Templ H, Spona J, Geyer G. Medroxyprogesterone acetate lowers plasma corticotropin and cortisol but does not suppress anterior pituitary responsiveness to human corticotropin releasing factor. Cancer. 1990; 66(9): 1949–53. 10.1002/1097-0142(19901101)66:9<1949::AID-CNCR2820660917>3.0.CO;2-E 2146010

[27] Dowsett M, Lal A, Smith IE, Jeffcoate SL. The effects of low and high dose medroxyprogesterone acetate on sex steroids and sex hormone binding globulin in postmenopausal breast cancer patients. Br J Cancer. 1987; 55(3): 311–3. 10.1038/bjc.1987.61 2952154 PMC2001763

[28] De Leo V, Morgante G, Piomboni P, Musacchio MC, Petraglia F, Cianci A. Evaluation of effects of an oral contraceptive containing ethinylestradiol combined with drospirenone on adrenal steroidogenesis in hyperandrogenic women with polycystic ovary syndrome. Fertil Steril. 2007; 88(1): 113–7. 10.1016/j.fertnstert.2006.11.137 17418832

[29] Kovalevsky G, Ballagh SA, Stanczyk FZ, Lee J, Cooper J, Archer DF. Levonorgestrel effects on serum androgens, sex hormone-binding globulin levels, hair shaft diameter, and sexual function. Fertil Steril. 2010; 93(6): 1997–2003. 10.1016/j.fertnstert.2008.12.095 19394598

[30] Agren UM, Anttila M, Maenpaa-Liukko K, Rantala ML, Rautiainen H, Sommer WF, Mommers E. Effects of a monophasic combined oral contraceptive containing nomegestrol acetate and 17beta-oestradiol in comparison to one containing levonorgestrel and ethinylestradiol on markers of endocrine function. Eur J Contracept Reprod Health Care. 2011; 16(6): 458–67. 10.3109/13625187.2011.614363 21942708 PMC3233273

[31] Payne AH, Hales DB. Overview of steroidogenic enzymes in the pathway from cholesterol to active steroid hormones. Endocr Rev. 2004; 25(6): 947–70. 10.1210/er.2003-0030 15583024

[32] Sanderson JT. The steroid hormone biosynthesis pathway as a target for endocrine-disrupting chemicals. Toxicol Sci. 2006; 94(1): 3–21. 10.1093/toxsci/kfl051 16807284

[33] Hu J, Zhang Z, Shen WJ, Azhar S. Cellular cholesterol delivery, intracellular processing and utilization for biosynthesis of steroid hormones. Nutrition and Metabolism. 2010; 7(47): 1–25. 10.1186/1743-7075-7-47 20515451 PMC2890697

[34] Lee TC, Miller WL, Auchus RJ. Medroxyprogesterone acetate and dexamethasone are competitive inhibitors of different human steroidogenic enzymes. J Clin Endocrinol Metab. 1999; 84(6): 2104–10. 10.1210/jcem.84.6.5646 10372718

[35] Satyaswaroop PG, Gurpide E. A direct effect of medroxyprogesterone acetate on 17 beta-hydroxysteroid dehydrogenase in adult rat testis. Endocrinology. 1978; 102(6): 1761–5. 10.1210/endo-102-6-1761 744049

[36] Barbieri RL, Ryan KJ. Direct effects of medroxyprogesterone acetate (MPA) and megestrol acetate (MGA) on rat testicular steroidogenesis. Acta Endocrinol (Copenh). 1980; 94(3): 419–25. 10.1530/acta.0.0940419 6968496

[37] Mizutani T, Sakata M, Miyake A, Tanizawa O, Terada N, Matsumoto K, Terakawa N. No inhibitory effects of gestrinone and medroxyprogesterone acetate on the estrogen production by ovaries of hypophysectomized rats stimulated by gonadotropins. Endocrinol Jpn. 1992; 39(6): 615–21. 10.1507/endocrj1954.39.615 1338193

[38] Arakawa S, Mitsuma M, Iyo M, Ohkawa R, Kambegawa A, Okinaga S, Arai K. Inhibition of rat ovarian 3 beta-hydroxysteroid dehydrogenase (3 beta-HSD), 17 alpha-hydroxylase and 17,20 lyase by progestins and danazol. Endocrinol Jpn. 1989; 36(3): 387–94. 2583058 10.1507/endocrj1954.36.387

[39] Overturf MD, Overturf CL, Carty DR, Hala D, Huggett DB. Levonorgestrel exposure to fathead minnows (Pimephales promelas) alters survival, growth, steroidogenic gene expression and hormone production. Aquat Toxicol. 2014; 148: 152–61. 10.1016/j.aquatox.2014.01.012 24503577

[40] Labrie F, Simard J, Luu-The V, Belanger A, Pelletier G. Structure, function and tissue-specific gene expression of 3beta-hydroxysteroid dehydrogenase/5-ene-4-ene isomerase enzymes in classical and peripheral intracrine steroidogenic tissues. J Steroid Biochem Mol Biol. 1992; 43(8): 805–26. 10.1016/0960-0760(92)90308-6 22217825

[41] Labrie F, Luu-The V, Lin SX, Labrie C, Simard J, Breton R, Belanger A. The key role of 17 beta-hydroxysteroid dehydrogenases in sex steroid biology. Steroids. 1997; 62(1): 148–58. 9029730 10.1016/s0039-128x(96)00174-2

[42] Payne AH, Abbaszade IG, Clarke TR, Bain PA, Park CH. The multiple murine 3 beta-hydroxysteroid dehydrogenase isoforms: structure, function, and tissue- and developmentally specific expression. Steroids. 1997; 62(1): 169–75. 9029733 10.1016/s0039-128x(96)00177-8

[43] Gazdar AF, Oie HK, Shackleton CH, Chen TR, Triche TJ, Myers CE, et al Establishment and characterization of a human adrenocortical carcinoma cell line that expresses multiple pathways of steroid biosynthesis. Cancer Res. 1990; 50(17): 5488–96. 2386954

[44] Rainey WE, Bird IM, Mason JI. The NCI-H295 cell line: a pluripotent model for human adrenocortical studies. Mol Cell Endocrinol. 1994; 100(1–2): 45–50. 10.1016/0303-7207(94)90277-1 8056157

[45] Hecker M, Newsted JL, Murphy MB, Higley EB, Jones PD, Wu R, Giesy JP. Human adrenocarcinoma (H295R) cells for rapid in vitro determination of effects on steroidogenesis: hormone production. Toxicol Appl Pharmacol. 2006; 217(1): 114–24. 10.1016/j.taap.2006.07.007 16962624

[46] Rijk JC, Peijnenburg AA, Blokland MH, Lommen A, Hoogenboom RL, Bovee TF. Screening for modulatory effects on steroidogenesis using the human H295R adrenocortical cell line: a metabolomics approach. Chem Res Toxicol. 2012; 25(8): 1720–31. 10.1021/tx3001779 22768806

[47] Schloms L, Storbeck KH, Swart P, Gelderblom WC, Swart AC. The influence of Aspalathus linearis (Rooibos) and dihydrochalcones on adrenal steroidogenesis: quantification of steroid intermediates and end products in H295R cells. J Steroid Biochem Mol Biol. 2012; 128(3–5): 128–38. 10.1016/j.jsbmb.2011.11.003 22101210

[48] Freshney RI, Culture of Animal Cells, A Manual of Basic Technique, Fourth ed, Wiley-Liss, New York, 1987.

[49] Bradford MM. A rapid and sensitive method for the quantitation of microgram quantities of protein utilizing the principle of protein-dye binding. Anal Biochem. 1976; 72: 248–54. 10.1016/0003-2697(76)90527-3 942051

[50] Verhoog NJ, Joubert E, Louw A. Evaluation of the phytoestrogenic activity of Cyclopia genistoides (honeybush) methanol extracts and relevant polyphenols. J Agric Food Chem. 2007; 55(11): 4371–81. 10.1021/jf063588n 17461595

[51] Quanson JL, Stander MA, Pretorius E, Jenkinson C, Taylor AE, Storbeck KH. High-throughput analysis of 19 endogenous androgenic steroids by ultra-performance convergence chromatography tandem mass spectrometry. J Chromatogr B Analyt Technol Biomed Life Sci. 2016; 1031: 131–138. 10.1016/j.jchromb.2016.07.024 27479683

[52] Hassan-Smith ZK, Morgan SA, Sherlock M, Hughes B, Taylor AE, Lavery GG, et al Gender-Specific Differences in Skeletal Muscle 11beta-HSD1 Expression Across Healthy Aging. J Clin Endocrinol Metab. 2015; 100(7): 2673–81. 10.1210/jc.2015-1516 25989394

[53] Wenzel J, Grabinski N, Knopp CA, Dendorfer A, Ramanjaneya M, Randeva HS, et al Hypocretin/orexin increases the expression of steroidogenic enzymes in human adrenocortical NCI H295R cells. Am J Physiol Regul Integr Comp Physiol. 2009; 297(5): R1601–9. 10.1152/ajpregu.91034.2008 19793950

[54] Hilscherova K, Jones PD, Gracia T, Newsted JL, Zhang X, Sanderson JT, et al Assessment of the effects of chemicals on the expression of ten steroidogenic genes in the H295R cell line using real-time PCR. Toxicol Sci. 2004; 81(1): 78–89. 10.1093/toxsci/kfh191 15187238

[55] Ishibashi H, Suzuki T, Suzuki S, Moriya T, Kaneko C, Takizawa T, et al Sex steroid hormone receptors in human thymoma. J Clin Endocrinol Metab. 2003; 88(5): 2309–17. 10.1210/jc.2002-021353 12727990

[56] Pfaffl MW. A new mathematical model for relative quantification in real-time RT-PCR. Nucleic Acids Res. 2001; 29(9): e45 10.1093/nar/29.9.e45 11328886 PMC55695

[57] Seamon KB, Padgett W, Daly JW. Forskolin: unique diterpene activator of adenylate cyclase in membranes and in intact cells. Proc Natl Acad Sci U S A. 1981; 78(6): 3363–7. 10.1073/pnas.78.6.3363 6267587 PMC319568

[58] Potts GO, Creange JE, Hardomg HR, Schane HP. Trilostane, an orally active inhibitor of steroid biosynthesis. Steroids. 1978; 32(2): 257–67. 10.1016/0039-128X(78)90010-7 715820

[59] Fuhrmann U, Krattenmacher R, Slater EP, Fritzemeier KH. The novel progestin drospirenone and its natural counterpart progesterone: biochemical profile and antiandrogenic potential. Contraception. 1996; 54(4): 243–51. 10.1016/S0010-7824(96)00195-3 8922878

[60] Krattenmacher R. Drospirenone: pharmacology and pharmacokinetics of a unique progestogen. Contraception. 2000; 62(1): 29–38. 10.1016/S0010-7824(00)00133-5 11024226

[61] Thomas JL, Mack VL, Sun J, Terrell JR, Bucholtz KM. The functions of key residues in the inhibitor, substrate and cofactor sites of human 3beta-hydroxysteroid dehydrogenase type 1 are validated by mutagenesis. J Steroid Biochem Mol Biol. 2010; 120(4–5): 192–9. 10.1016/j.jsbmb.2010.04.015 20420909 PMC2891085

[62] Thigpen JT, Brady MF, Alvarez RD, Adelson MD, Homesley HD, Manetta A, et al Oral medroxyprogesterone acteate in the treatment of advanced or recurrent endometrial carcinoma: a dose-response study by the gynecologic oncology group. J Clin Oncol. 1999; 17: 1736–1744. 10561210 10.1200/JCO.1999.17.6.1736

[63] Focan C, Beauduin M, Salamon E, de Greve J, de Wasch G, Lobelle JP, et al Adjuvant high-dose medroxyprogesterone acetate for early breast cancer: 13 years update in a multicentre randomized trial. Br J Cancer. 2001; 85(1): 1–8. 10.1054/bjoc.2001.1829 11437394 PMC2363916

[64] Ito F, Mori T, Takaoka O, Tanaka Y, Koshiba A, Tatsumi H, et al Effects of drospirenone on adhesion molecule expression and monocyte adherence in human endothelial cells. Eur J Obstet Gynecol Reprod Biol. 2016; 201: 113–7. 10.1016/j.ejogrb.2016.03.044 27088625

[65] Bahamondes L, Bahamondes MV. New and emerging contraceptives: a state-of-the-art review. Int J Womens Health. 2014; 6: 221–34. 10.2147/IJWH.S46811 24570597 PMC3933723

[66] Gerrits MG, Schnabel PG, Post TM, Peeters PA. Pharmacokinetic profile of nomegestrol acetate and 17beta-estradiol after multiple and single dosing in healthy women. Contraception. 2013; 87(2): 193–200. 10.1016/j.contraception.2012.07.001 22898360

[67] Blode H, Kowal K, Roth K, Reif S. Pharmacokinetics of drospirenone and ethinylestradiol in Caucasian and Japanese women. Eur J Contracept Reprod Health Care. 2012; 17(4): 284–97. 10.3109/13625187.2012.677076 22680989 PMC3439798

[68] Massai MR, Diaz S, Quinteros E, Reyes MV, Herreros C, Zepeda A, et al Contraceptive efficacy and clinical performance of Nestorone implants in postpartum women. Contraception. 2001; 64(6): 369–76. 10.1016/S0010-7824(01)00259-1 11834236

[69] Brache V, Mishell DR, Lahteenmaki P, Alvarez F, Elomaa K, Jackanicz T, Faundes A. Ovarian function during use of vaginal rings delivering three different doses of Nestorone. Contraception. 2001; 63(5): 257–61. 10.1016/S0010-7824(01)00199-8 11448466

[70] Robbins A, Bardin CW. Nestorone progestin. The ideal progestin for use in controlled release delivery systems. Ann N Y Acad Sci. 1997; 828: 38–46. 9329822 10.1111/j.1749-6632.1997.tb48522.x

[71] Blode H, Foidart JM, Heithecker R. Transfer of drospirenone to breast milk after a single oral administration of 3 mg drospirenone + 30 microg ethinylestradiol to healthy lactating women. Eur J Contracept Reprod Health Care. 2001; 6(3): 167–71. 11763981

[72] Berrino F, Muti P, Micheli A, Bolelli G, Krogh V, Sciajno R, et al Serum sex hormone levels after menopause and subsequent breast cancer. J Natl Cancer Inst. 1996; 88(5): 291–6. 10.1093/jnci/88.5.291 8614008

[73] Key T, Appleby P, Barnes I, Reeves G. Endogenous sex hormones and breast cancer in postmenopausal women: reanalysis of nine prospective studies. J Natl Cancer Inst. 2002; 94(8): 606–16. 11959894 10.1093/jnci/94.8.606

[74] Eliassen AH, Missmer SA, Tworoger SS, Hankinson SE. Endogenous steroid hormone concentrations and risk of breast cancer: does the association vary by a woman's predicted breast cancer risk? J Clin Oncol. 2006; 24(12): 1823–30. 10.1200/JCO.2005.03.7432 16567770

[75] Dorgan JF, Stanczyk FZ, Kahle LL, Brinton LA. Prospective case-control study of premenopausal serum estradiol and testosterone levels and breast cancer risk. Breast Cancer Res. 2010; 12(6): R98 10.1186/bcr2779 21087481 PMC3046441

[76] Birrell SN, Butler LM, Harris JM, Buchanan G, Tilley WD. Disruption of androgen receptor signaling by synthetic progestins may increase risk of developing breast cancer. FASEB J. 2007; 21(10): 2285–93. 10.1096/fj.06-7518com 17413000

[77] Proverbs-Singh T, Feldman JL, Morris MJ, Autio KA, Traina TA. Targeting the androgen receptor in prostate and breast cancer: several new agents in development. Endocr Relat Cancer. 2015; 22(3): R87–R106. 10.1530/ERC-14-0543 25722318 PMC4714354

[78] Fournier A, Berrino F, Riboli E, Avenel V, Clavel-Chapelon F. Breast cancer risk in relation to different types of hormone replacement therapy in the E3N-EPIC cohort. Int J Cancer. 2005; 114(3): 448–54. 10.1002/ijc.20710 15551359

