# Supplementary figures and images for: Fourth-Generation Progestins Inhibit 3β-Hydroxysteroid Dehydrogenase Type 2 and Modulate the Biosynthesis of Endogenous Steroids
Source: PLoS One. 2016 Oct 5;11(10):e0164170. doi: 10.1371/journal.pone.0164170 (PMC5051719; doi:10.1371/journal.pone.0164170)

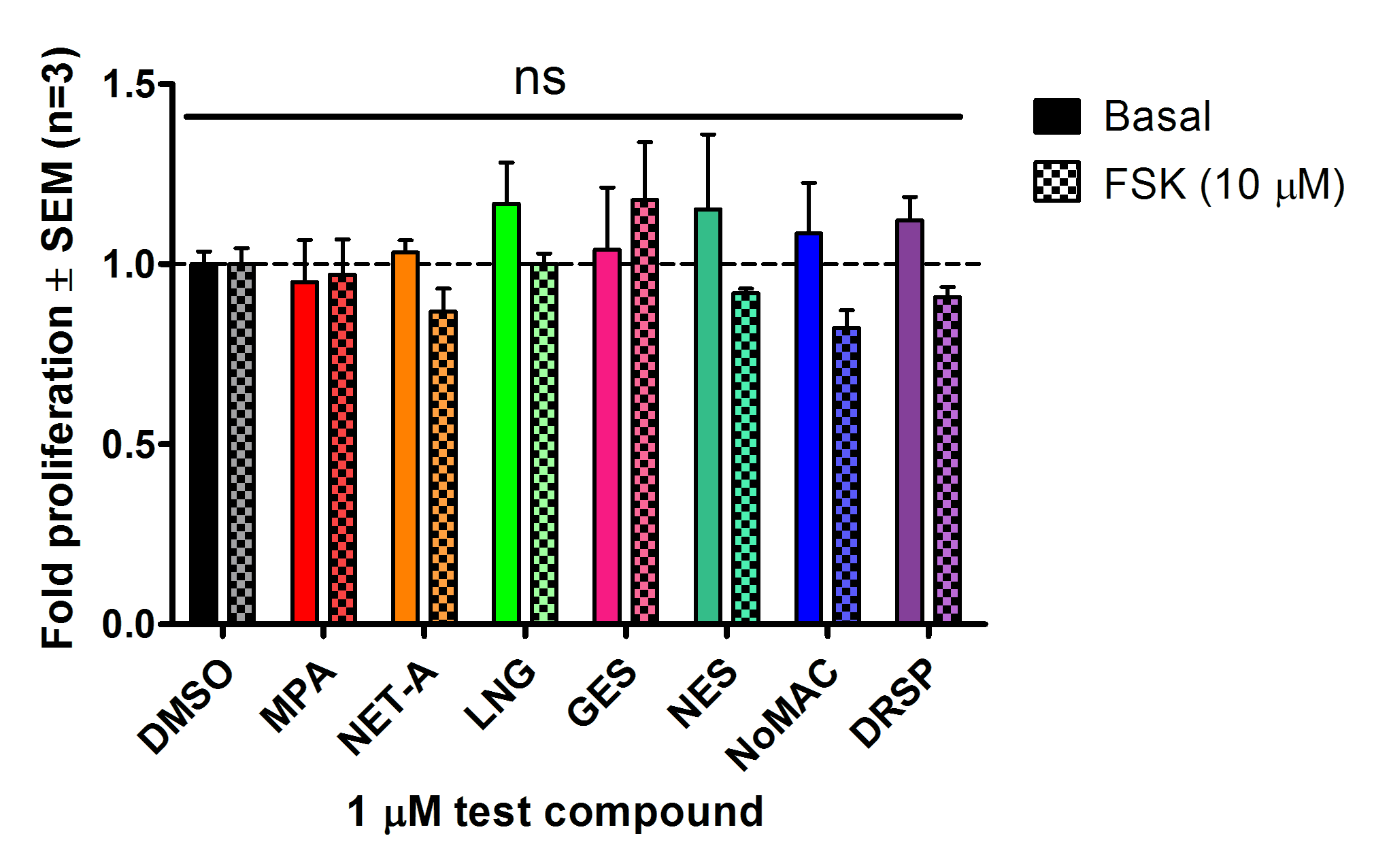

Supplement: S1 Fig — Cells were incubated for 48 hours with DMSO (vehicle control) or 1 μM MPA, NET-A, LNG, GES, NES, NoMAC or DRSP in the absence or presence of 10 μM FSK. Cell viability was measured using the MTT assay and results are expressed as fold proliferation relative to DMSO = 1. Results shown are the average of three independent experiments (±SEM) performed in triplicate. (TIF) [file pone.0164170.s001.tif]

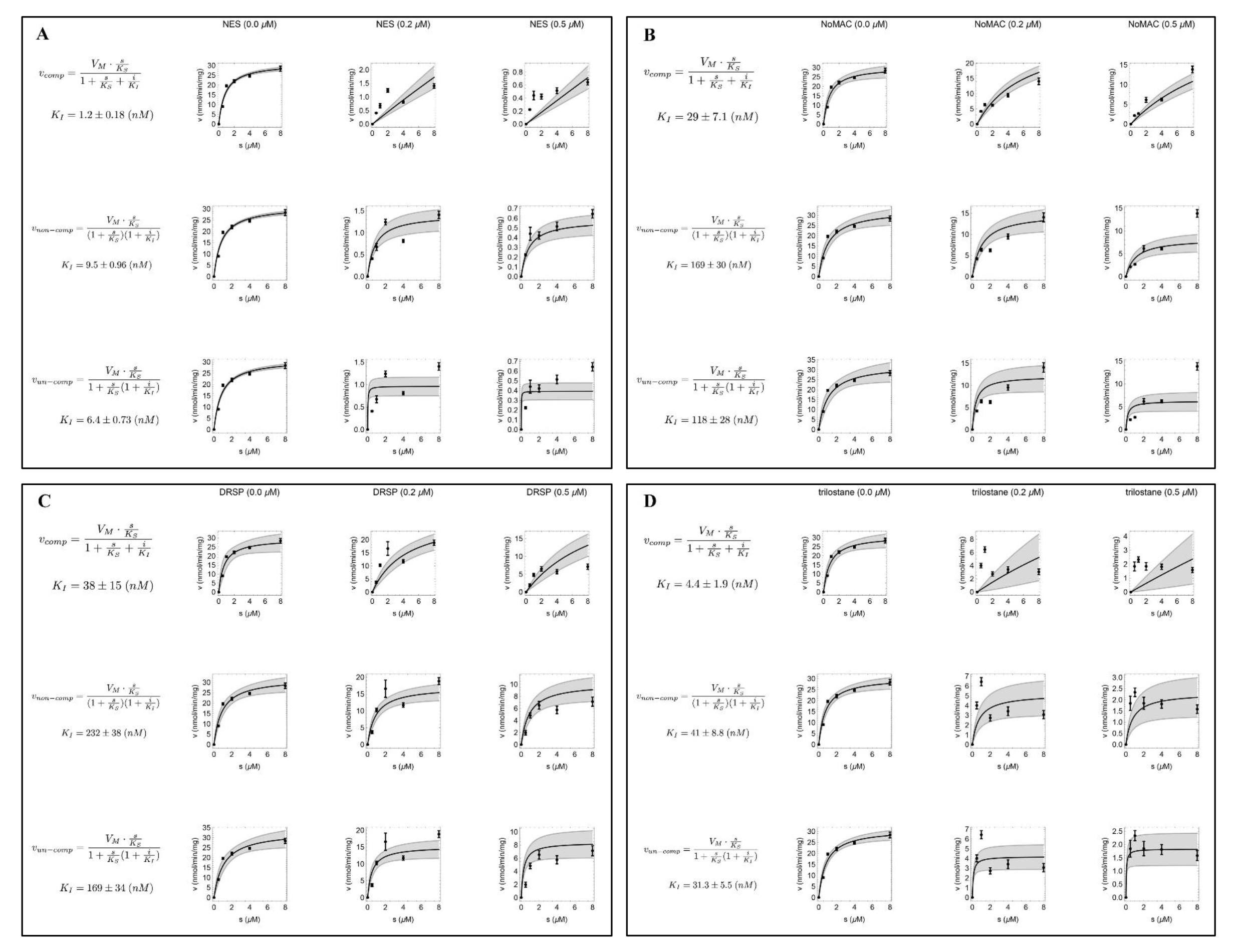

Supplement: S2 Fig — COS-1 cells were transiently transfected with a plasmid expressing human 3βHSD2 (pCDNA6-hHSD3β2-V5), and subsequently treated with Preg (0.5, 1, 2, 4 and 8 μM) in the presence of 0.0, 0.2 or 0.5 μM NES, NoMAC, DRSP or trilostane. The conversion of Preg to Prog was analyzed using UPLC-MS/MS. Three inhibitory mechanisms were fitted to the data sets: competitive, non-competitive and uncompetitive, using the rate equations shown in the figure. Confidence intervals (95%) for the fits are indicated in the plots with grey fillings. Each data point represents the mean ± SE of at least duplicate experiments. (TIF) [file pone.0164170.s002.tif]

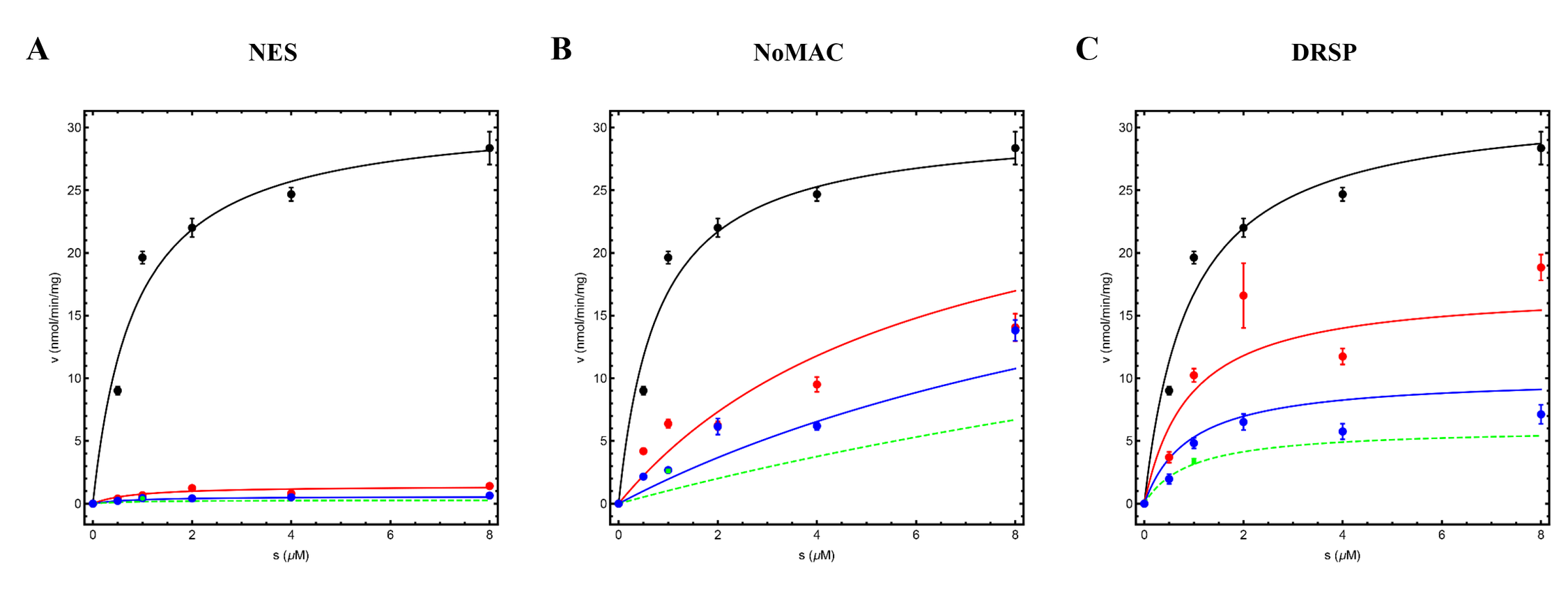

Supplement: S3 Fig — Michaelis-Menten plots were predicted (dashed green line) based on the data presented in Fig 6. The predicted Vmax in the presence of 1 μM NES (0.16 ± 0.03 nmol/min/mg), NoMAC (1.04 ± 0.30 nmol/min/mg) and DRSP (3.16 ± 0.87 nmol/min/mg) correlates with the residual activities determined experimentally (NES, 0.43 ± 0.01 nmol/min/mg; NoMAC, 2.62 ± 0.11 nmol/min/mg; DRSP, 3.39 ± 0.19 nmol/min/mg) as shown in Fig 5A. (TIF) [file pone.0164170.s003.tif]
