# Supplementary material for: Fourth-Generation Progestins Inhibit 3β-Hydroxysteroid Dehydrogenase Type 2 and Modulate the Biosynthesis of Endogenous Steroids
Source: PLoS One. 2016 Oct 5;11(10):e0164170. doi: 10.1371/journal.pone.0164170 (PMC5051719; doi:10.1371/journal.pone.0164170)
Supplement: S1 Table — (PDF) [file pone.0164170.s004.pdf]

Steroid metabolite	Steroid production (µM/mg protein) ± SEM		Fold change ± SEM		
	Basal	+ FSK			
Preg	0.20 ± 0.02	1.85 ± 0.10	↑	9.06 ± 1.24	***
Prog	0.02 ± 0.00	0.07 ± 0.01	↑	4.00 ± 0.97	***
17OH-Prog	0.03 ± 0.01	0.08 ± 0.01	↑	2.90 ± 0.04	***
16OH-Prog	0.03 ± 0.02	0.08 ± 0.05	↑	2.83 ± 0.09	**
DOC	0.62 ± 0.10	5.01 ± 0.87	↑	8.20 ± 1.03	***
CORT	0.16 ± 0.04	3.03 ± 0.61	↑	13.63 ± 1.76	***
11-DHC	0.003 ± 0.000	0.01 ± 0.00	↑	1.79 ± 0.20	*
Ald	0.00 ± 0.00	0.01 ± 0.00	↑	5.59 ± 1.84	***
Deoxycortisol	1.92 ± 0.39	4.64 ± 1.18	↑	2.78 ± 0.56	**
Cortisol	0.17 ± 0.07	1.34 ± 0.25	↑	6.24 ± 0.16	***
Cortisone	0.00 ± 0.00	0.00 ± 0.00		-	
DHEA	0.05 ± 0.03	0.54 ± 0.26	↑	10.38 ± 2.04	***
A4	0.34 ± 0.04	0.46 ± 0.16		-	
11OH-A4	0.01 ± 0.01	0.03 ± 0.02	↑	4.41 ± 1.29	**
Testosterone	0.012 ± 0.00	0.02 ± 0.01	↓	1.33 ± 0.03	ns
**Total steroid**	**3.63 ± 0.72**	**15.55 ± 1.52**	↑	**4.29 ± 0.62**	**

The human H295R cell line was incubated with DMSO (vehicle control) in the absence and presence of forskolin (FSK) for 48 hours. Steroids were extracted and quantified by UPLC–MS/MS. The concentration of the total steroids produced (µM) were normalized to protein concentration (mg/ml). Results indicate the total steroid production (µM/mg protein) under basal and FSK-stimulated conditions, as well as the fold change in response to FSK treatment (calculated relative to basal, which was set as 1). Results shown are the average of three independent experiments with each condition performed in triplicate (±SEM). (-) denotes no effect; 17OH-Preg, DHT, estrone and 17β-estradiol could not be detected with the current method.
